# Non-steroidal Anti-inflammatory Drug (NSAID)-, Potassium Supplement-, Bisphosphonate-, and Doxycycline-Mediated Peptic Ulcer Effects: A Narrative Review

**DOI:** 10.7759/cureus.51894

**Published:** 2024-01-08

**Authors:** Camryn L Keller, Nicholas T Jones, Raegan B Abadie, William Barham, Raju Behara, Shilpadevi Patil, Antonella Paladini, Shahab Ahmadzadeh, Sahar Shekoohi, Giustino Varrassi, Alan D Kaye

**Affiliations:** 1 School of Medicine, Louisiana State University Health Sciences Center, Shreveport, USA; 2 Department of Anesthesiology, Louisiana State University Health Sciences Center, Shreveport, USA; 3 Department of Life, Health and Environmental Sciences (MESVA), University of L'Aquila, L'Aquila, ITA; 4 Department of Pain Medicine, Paolo Procacci Foundation, Rome, ITA

**Keywords:** doxycycline, bisphosphonates, potassium, non-steroidal anti-inflammatory drugs (nsaids), peptic ulcers

## Abstract

Peptic ulcers are a common condition that arises from an imbalance between acid production and gastroduodenal protective factors. Various drugs, including non-steroidal anti-inflammatory drugs (NSAIDs), potassium supplements, bisphosphonates, and doxycycline, can increase the development of peptic ulcers. NSAIDs are one of the most common medications prescribed for pain relief, and they also inhibit the formation of cyclooxygenase-1 (COX-1). COX-1 helps in the production of mucus that lines the stomach, so by inhibiting COX-1, NSAIDs reduce the mucus produced by the stomach and increase the likelihood of gastric ulcer formation. Additionally, NSAIDs are acidic, and increasing the amount of any acid in the stomach can result in promoting ulcer development. Potassium supplements are used to reduce the effects of hypertension, decrease the development of kidney stones, and treat hypokalemia. The various types of transporters and channels used to move potassium across cell membranes increase hydrogen being pumped, increasing gastric acid production and ulcer formation. Bisphosphonates are used to treat a variety of skeletal disorders that require inhibition of osteoclast activity. Nitric oxide (NO) has been shown to have a therapeutic effect on gastric ulcers, and some bisphosphonates have been shown to decrease the production of nitric oxide, resulting in increased damage to the gastric mucosa. Finally, doxycycline is a broad-spectrum tetracycline antibiotic that is typically used to treat anthrax poisoning, skin lesions, and sexually transmitted diseases. A harmful adverse effect of doxycycline is the formation of peptic and gastric ulcers related to the drug being highly acidic once it has dissolved.

## Introduction and background

Peptic ulcers are extremely common in the United States, with approximately four million people having an ulcer and over 350,000 new cases being reported annually [1]. Although this is a very high number of new case reports, this number has decreased in recent years, and this is mostly due to new treatment options. Peptic ulcers and complications associated with them have been shown to have a significant impact on the US economy, with the total cost being about $5.65 billion each year [2]. Studies have shown that peptic ulcers are more common in males overall, and incidence rates increase in the 50-59 year age range but peak in the 60-69 year age range [3]. Additionally, there is a genetic factor in the development of peptic ulcers, which are 2-3 times more common in patients with first-degree relatives who also have peptic ulcers [1]. There are multiple causes of peptic ulcers, with the most common being Helicobacter pylori infection and ingestion of medications, especially non-steroidal anti-inflammatory drugs (NSAIDs) [4]. Other risk factors for peptic ulcers include smoking and other chronic medical conditions, and studies have shown that peptic ulcers are also more common in low-income countries [5,6]. A physiological balance between the pepsin within gastric acid and the gastroduodenal defense system is necessary to protect the gastric mucosa. If this balance is disrupted, the gastric acid may damage the mucosa. The acid can erode the mucosa until it breaks, which causes ulcerations, most commonly occurring in the stomach and proximal duodenum [7,8]. However, increases in acid production of destruction of the mucosa can occur throughout the gastrointestinal system, including the esophagus, and this forms a peptic ulcer as opposed to a gastric ulcer, which forms in the stomach. Diagnosis of peptic ulcer includes testing for H. pylori infection in areas with a high prevalence of the bacteria or in patients without major symptoms, and in patients with symptoms, diagnosis is done by upper GI endoscopy [6]. The most common symptoms of peptic ulcers are epigastric pain, dyspepsia, abdominal fullness, and early satiety, and these symptoms may be constant or episodic [6]. However, elderly patients may be asymptomatic, or symptoms may be extremely mild compared to younger patients. The major complication of peptic ulcer is bleeding, which may present with melena, hematemesis, or iron deficiency anemia, and other complications of gastric ulcers including perforation and gastric outlet obstruction [4,6]. Additionally, studies have shown that the recurrence rates for these complications within a one-month period can be over 35% in some populations [2]. There have been major developments in treatment for peptic ulcers, such as proton pump inhibitors, a type of medication used to decrease gastric acid production, allowing the ulcer to heal. Additionally, patients whose peptic ulcer is due to an H. pylori infection are given antibiotics, and patients taking medications causing peptic ulcers are advised to stop taking the offending medication [6]. Various medications such as NSAIDs, potassium supplements, bisphosphonates, and doxycycline have been shown to increase the risk and prevalence of peptic ulcers in patients taking them. This occurs through various mechanisms that will be explored throughout this review.

## Review

Non-steroidal anti-inflammatory drugs

Mechanism of Action and Uses

NSAIDs are some of the most common over-the-counter medications for many forms of acute pain management. NSAIDs are used for symptomatic control of pain and inflammation in many chronic musculoskeletal autoimmune conditions, such as rheumatoid arthritis, osteoarthritis, spondylarthritis, and more [9,10]. This pain control is mediated through a few different mechanisms. Overall, NSAIDs exert their pain-blocking effects by blocking prostaglandins (PGs), which are typically responsible for pain. PGs are produced in response to pain and inflammatory stimuli, and the pathway starts with the phospholipids found on the cell membrane of cells. The enzyme phospholipase A2 takes that cell membrane phospholipids and catalyzes the reaction to convert it to arachidonic acid, the first step in the committed pathway for PG synthesis. Arachidonic acid is then converted by a group of cyclooxygenases (COX) enzymes that determine their fate and functionality. There are two major isotypes of the COX enzymes, COX-1 and COX-2 [9]. COX-1 is expressed constitutively under basal conditions because it is involved in homeostatic pathways such as platelet aggregation, vascular homeostasis, kidney function, and the production of mucus. COX-2 is expressed at very low levels in most tissues and promotes the inflammatory pathway, and its expression is rapidly increased at sites of stress, pain, and tissue damage or irritation [11]. Either of these COX enzymes converts the arachidonic acid to prostaglandin G2 (PGG2), which then undergoes a reduction process to become the unstable intermediate prostaglandin H2 (PGH2). After the production of PGH2, the molecule rapidly undergoes conversion into different tissue-specific PG molecules with the assistance of terminal synthase enzymes. These molecules are thromboxane A2 (TAX), prostaglandin F2 - alpha (PGF2α), prostaglandin E2 (PGE2), prostaglandin D2 (PGD2), and prostaglandin I2 (PGI2). The final amount of each different PG produced is affected by the relative levels of expression of COX-1 and COX-2 and terminal synthases enzymes. The overall mechanism of action of NSAIDs is inhibition of the COX enzymes in the aforementioned PG synthesis pathways [9].

NSAIDs are commonly classified into four broader classes that include carboxylic acids, enolic acids, acidic, or COX-2 selective. Under the carboxylic acid class, the subclasses include salicylic acid, acetic acids, propionic acids, and fenamic acids. Acetylsalicylic acid (ASA), more commonly known as aspirin, falls under the salicylic acid subclass and inhibits the pathway by covalently and irreversibly binding to the COX enzymes, effectively rendering them inactive for the lifespan of that enzyme [9]. Low-dose ASA reduces the risk of cardioembolic events in patients with comorbidities, such as atrial fibrillation, due to the irreversible inhibition and antiplatelet effects [12]. All the other medications classified as NSAIDs are competitive inhibitors of the COX system, where they compete for the active binding site of the COX enzyme with arachidonic acid. The COX-2 selective class of medications, which include celecoxib (Celebrex), etoricoxib (Arcoxia), and lumiracoxib (Prexige), solely inhibit the COX-2 enzyme [9]. The development of selective COX-2 inhibitors has alleviated some of the most commonly reported side effects, such as destruction of cartilage, hepato-renal dysfunction, and organ failure when taking non-selective NSAIDs [13].

Side Effects

NSAIDs have a constellation of side effects due to their COX-blocking abilities. Most NSAIDs are negatively linked to cardiovascular diseases such as myocardial infarction or stroke, with a notable exception being the coxib family of NSAIDs. One study on 8067 participants showed that rofecoxib has been linked to a fivefold increase in the risk of myocardial infarction (MI) or stroke when compared to treatment with naproxen in patients with rheumatoid or osteoarthritis [14,15]. Most NSAIDs carry the risk of renal injury most notably in the form of acute kidney injury (AKI) and renal papillary necrosis. Approximately 15% of AKI has been associated with the use of NSAIDs and with several electrolyte imbalances [15]. Among all side effects, the gastrointestinal side effects have been well elucidated in the literature [16,17]. 

NSAIDs with the most potent COX-1 inhibition, such as ASA, indomethacin, and piroxicam, have been shown to produce the most damage to the stomach mucosa. As previously discussed, the pathway of COX-1 is involved in the production of mucus that lines the stomach; therefore, inhibition of the COX-1 enzyme leads to less mucosal protection and, thus, the production of a higher level of gastrointestinal toxicity [16]. NSAIDs are weakly acidic in their structure, which allows them to easily cross the mucosal layer and become ionized in the neutral pH of the surrounding stomach cells, where they exert their effects [7]. According to a study by Arnal et al., upper GI lesions can be found in up to 50% of patients on chronic NSAID therapy and 30- 40% of patients had gastric erosions or ulcers. There is an increased risk of peptic ulcers that is four times greater in patients on NSAIDs when compared to control populations. Patients who are required to be on chronic NSAID management and have an increased risk of peptic ulcers should apply preventative measures to reduce the risk of peptic ulcer disease [17]. The first preventative measure is switching to a selective COX-2 inhibitor, if possible. Some studies have found that high-dose misoprostol (1,200 µg), a PG analog, has gastroprotective effects in anemic patients, while lower dosages (800 µg) lowered the permeability of indomethacin and protected the gastric lining [18].

Potassium supplements

Potassium Supplementation Uses

Potassium homeostasis is important for the body to function. Potassium is abundant in various foods, including fruits, beans, milk, vegetables, and cereals. Patients who have hypokalemia, or low serum potassium, can receive potassium supplementations in hopes of increasing their serum potassium levels. Potassium can also be combined with other metabolites, like citrate or chloride, to have different clinical effects. Potassium has a role in the transmission of nerve signals, contraction of vascular smooth muscle, and fluid maintenance via aldosterone [19-21]. Potassium supplements can be used for the treatment of high blood pressure, help increase low potassium levels, and in the prevention of stroke [22]. The World Health Organization, WHO, suggests a potassium intake of at least 3.5 g/day from food for adults to reduce a variety of cardiovascular symptoms.

In essential hypertension, potassium supplements can reduce systolic and diastolic blood pressure in patients with or without hypertension, making it an effective antihypertensive for essential hypertension as long as it doesn't exceed the daily recommended dietary intake recommendations [23]. The Dietary Approaches to Stop Hypertension eating pattern focuses on patients getting more potassium out of their diet than they normally would, which can reduce blood pressure. This diet is often recommended to patients with hypertension, as a pre-medical intervention to try to reduce their high blood pressure [24]. 

Potassium supplements can also be used in individuals who commonly develop kidney stones. This is because low potassium can decrease calcium reabsorption in the kidneys, increasing the risk of developing elevated urine calcium, or hypercalciuria, and this often advances into kidney stones. It has also been found that potassium can protect bone through its acid-base equilibrium by producing an alkaline component from the potassium salts, which can help protect the bone tissue. Individuals with diabetes can also be affected by low potassium levels, so it is important to have type 2 diabetics receive potassium supplements. This is because potassium is essential for the pancreatic beta cells to secrete insulin, to in turn decrease glucose. Without potassium, one cannot secrete insulin, leading to hyperglycemia and worsening diabetes [24].

Side Effects

Potassium channels can increase gastric acid secretion, which can, in turn, lead to an increase in ulcerations. Therefore, many medications used for peptic ulcer disease are called "proton pump inhibitors," which inhibit potassium pumps. These potassium pumps can activate the H/K ATPase which can result in the secretion of hydrochloric acid into the parietal cell, furthering acidic synthesis and increasing the risk for ulcers. It has been shown that patients taking potassium supplementation medications, specifically for the treatment of hypokalemia, often result in the production of gastric ulcers as a side effect. 

One specific drug, Nicorandil, used for the treatment of ischemic heart disease, can stimulate nitrate and potassium ATP channels, and in turn, a side effect of GI, oral, and anal ulcers has been reported. Longer and more chronic use of potassium supplements has been shown with worse side effects, including ulcers in areas other than the GI tract [25].

Other side effects of potassium, besides gastric ulcers, include stomach upset, nausea, diarrhea, or vomiting. Potassium supplementation should not be used in patients with a history of kidney disease and should be closely monitored if the patient is on angiotensinogen-converting enzyme inhibitors, angiotensin receptor blockers, or potassium-sparing diuretics [22].

Bisphosphonates

Bisphosphonate Mechanism of Action and Uses

Bisphosphonates, a class of drugs structurally analogous to inorganic pyrophosphate, are used extensively in the treatment of osteoporosis, as well as in the management of osteogenesis imperfecta, metastatic bone disease, and other skeletal disorders [26-28]. As a chelator of positive divalent ions, bisphosphonates attach to the surface of bone, where they are taken up by osteoclasts and subsequently inhibit their function in bone breakdown [29]. Among bisphosphonates, nitrogen-containing bisphosphonates such as alendronate, risedronate, pamidronate, and zoledronic acid have higher relative potency in osteoclast inhibition, while bisphosphonates not containing a nitrogen side chain, such as etidronate, clodronate, and tiludronate, are less potent [30]. While dosage efficacy varies with the specific patient, oral bisphosphonates at 70mg once a week show similar tolerability and greater convenience versus a 10mg daily dose in older female patients with osteoporosis; bisphosphonates may also be administered intravenously [31].

Bisphosphonates are renally excreted, which makes them potentially nephrotoxic depending on the maximum serum concentration achieved [32]. Although bisphosphonates are generally well tolerated, IV bisphosphonates have been linked with acute phase reactions and oral bisphosphonates have been associated with gastrointestinal side effects depending on the dosing schedule and the amount of the dose, because an increased risk dose is associated with an increased risk of adverse effects [33]. Another more rare side effect of bisphosphonate treatment is osteonecrosis of the jaw [34].

Side Effects

Among bisphosphonates used for primary osteoporosis, zoledronic acid is thought to be the most likely to cause an adverse gastrointestinal event, while etidronate is the most likely to be discontinued due to gastrointestinal side effects [35]. With respect to risedronate and alendronate compared to placebo, other retrospective studies have found no clinically significant differences in adverse gastrointestinal effects [36]. However, other studies have found a significantly increased risk of the development of GI bleeding for patients taking alendronate, with a hazard ratio of 1.32 for the development of upper gastrointestinal bleeds and 1.84 for lower gastrointestinal bleeds for a population-representative cohort in Taiwan [37].

One proposed mechanism by which bisphosphonates may cause gastrointestinal damage is via the reduction in nitric oxide generation by nitric oxide synthase, as well as interfering with cell migration to sites of mucosal damage [38]. In experimental rat models, alendronate has been used to induce antral ulcers separate from prostaglandin depletion via NSAIDs, suggesting that the role of nitric oxide is a separate but potent mediator of gastrointestinal damage [39]. Supporting this synergistic relationship between gastrointestinal damage and ulcer formation, another study suggests that in Rheumatoid Arthritis patients, cotreatment with NSAIDS and bisphosphonates may act as separate risk factors for the development of peptic ulcers [40]. Another group of drugs that may synergistically increase peptic ulcer risk when co-administered with bisphosphonates are direct oral anticoagulants [41]. Rebamipide, an amino acid derivative acting as a mucosal protective agent, has been proposed as a prophylactic treatment for gastric ulcers caused by bisphosphonates due to its anti-inflammatory and antioxidative effects [42,43]. In summary, bisphosphonates seem to exhibit a little but clinically significant effect on the development of gastric ulcers via nitric oxide depletion, and without concomitant administration of another drug that irritates the gastric mucosa, bisphosphonates are generally well tolerated with respect to the gastrointestinal system.

Doxycycline

Mechanism of Action and Uses

Doxycycline is a second-generation tetracycline antibiotic that is considered a broad-spectrum agent, working on gram-positive and gram-negative bacteria, atypical organisms, and parasites [44]. This drug can also be used in the treatment or prevention of anthrax, a common septicemia [45]. Doxycycline works by inhibiting protein synthesis of the organism via inhibition of the aminoacyl-tRNA to the ribosomal acceptor (A) site. Without protein synthesis, the organism cannot replicate and continue to invade the host. Doxycycline is known as a bacteriostatic drug because of its reversibility in the prevention of the growth of bacteria [44]. Doxycycline has a high bioavailability and a long elimination half-life, which means you only have to give one or two daily oral doses. Doxycycline is often used as a first line for sexually transmitted infections, like syphilis, chlamydiosis, gonorrhea, and Mycoplasma genitalium [46].

Side Effects

A well-accepted side effect of oral doxycycline use is pill-induced esophagitis because when the pill dissolves, it creates an acidic environment in the esophagus. Pill-induced esophagitis can present as chest pain and odynophagia (painful swallowing) in patients with recent infection [47,48]. Esophageal ulceration is much more common than gastric ulcers caused by this medication; however, both are serious adverse events from oral use [49]. Esophageal ulcers can be minimized by drinking at least 100ml of water and swallowing the pill in an upright position [50]. One study showed that gastrointestinal side effects are almost inevitable when the dosage of medication reaches 200mg daily [51]. Once identified as a peptic ulcer caused by doxycycline use, the treatment involves removal of the offending agent and gastric acid inhibition. Most patients heal within 2-4 weeks after the necessary steps have been taken and serious complications such as esophageal perforation or strictures are extremely rare [49]. The correlations between various drugs and gastric erosion and ulcers are summarized in Table 1.

**Table 1:** Correlation Between Various Drugs and Gastric Erosion and Ulcers NSAIDs: Non-steroidal anti-inflammatory drugs; GI: gastrointestinal

Study	Groups Studied and Intervention	Results and Findings	Conclusions
Study 1: Non-steroidal anti-inflammatory drugs and life-threatening complications of peptic ulceration [52]	235 patients with life-threatening complications due to peptic ulcers were studied to determine how many of them used NSAIDs	60% of patients with bleeding and perforated ulcers were taking NSAIDs, and approximately 80% of ulcer-related deaths occurred in patients who were taking NSAIDs.	There is a strong correlation between patients taking NSAIDs and both the occurrence of peptic ulcers and peptic ulcer-related complications such as bleeding.
Study 2: Effect of potassium chloride supplements on upper gastrointestinal mucosa [53]	225 patients were given various forms of potassium supplementation, and the long-term adverse effects were studied	Among patients who received the potassium supplement with glycopyrrolate, 51% developed erosions and 11% developed ulcers. Among patients who received only the potassium supplement, 33% developed erosion, and 5% developed ulcers.	There is a significantly high correlation between potassium supplementation and erosion of gastric tissue, which can eventually result in ulceration. Some preparations of potassium supplementation may have a larger increase in ulcerative events compared to other preparations.
Study 3: Alendronate, a bisphosphonate, increased upper and lower gastrointestinal bleeding: risk factor analysis from a nationwide population-based study [37]	3,000 patients taking alendronate and 12,000 age and sex-matched controls were compared to determine the rate of GI bleeding, which was typically due to the development of peptic ulcers.	The rate of upper GI bleeding was 2.93% and lower GI bleeding was 2.17% in patients taking alendronate, which is significantly higher than the control group. This was determined after adjusting for potential confounding variables.	The rate of developing both upper and lower GI bleeding is significantly increased in patients taking alendronate compared to a similar population not taking the drug.
Study 4: Doxycycline-induced gastrointestinal injury [48]	There are three case reports of patients having doxycycline-induced gastrointestinal injury leading to erosion.	All three patients had endoscopic and histologic changes consistent with GI injury.	Gastrointestinal injury is a possible adverse effect of taking doxycycline.

Discussion

In the United States, peptic ulcers are a very common condition, with the most common population being males over the age of 60 [1,3]. Ulcers develop when there is an imbalance between the amount of gastric acid and the gastroduodenal defense system, which functions to protect the gastric mucosa from the acid. Gastric acid is able to erode the mucosa and cause an ulcer, and this occurs when there is either excess gastric acid or the gastroduodenal protective system is lost. Epigastric pain, dyspepsia, abdominal fullness, and early satiety are some of the most common symptoms associated with peptic ulcers; however, some may be asymptomatic, especially in older patients. Additionally, the ulcer may completely perforate, causing bleeding, melena, hematemesis, or iron deficiency anemia, and this is a very serious complication associated with peptic ulcers.

NSAIDs damage the gastroduodenal protective system by inhibiting the synthesis of COX-1. This reduces the production of a gastric mucosal layer, which increases the likelihood of ulcer formation. Additionally, potassium supplements increase peptic ulcer formation because when potassium is transported across the cell membrane of the stomach, more gastric acid is produced, overwhelming the gastroduodenal protective system. Another class of drugs that can result in gastric ulcers is bisphosphonates, which reduce the activity of nitric oxide synthase. This decreases the production of nitric oxide, which has been shown to help heal gastric ulcers. Additionally, bisphosphonates reduce the migration of cells useful in healing the ulcer, furthering the reduction in ulcer healing. Finally, an adverse effect of doxycycline is an increased risk for the formation of various types of peptic ulcers, primarily located in the esophagus, due to the drug being acidic once it has dissolved. The increase in acid is able to increase the destruction of mucosa throughout the gastrointestinal system.

## Conclusions

Although each of these medications has been proven effective in treating certain medical conditions, they all have an increased risk for peptic ulcer formation. When using or prescribing these drugs, the benefits of their effectiveness should be weighed against the possibility of peptic ulcer formation and the complications associated with peptic ulcers.
